# Notes of Cases

**Published:** 1853-07

**Authors:** Geo. W. Brown

**Affiliations:** Port Carbon, Pa.


*Notes of Cases.* By Geo. W. Brown, M. D., Port Carbon, Pa.

CASE I.—STRANGULATED HERNIA.

I was called to see Mrs. H., a tall, spare woman, 59 years of age, a native of Scotland, January 9th, 1852. I found her complaining of severe pain in the right groin, and extending from that point over the whole abdomen; a constant desire to go to stool without being able to pass anything from the bowels; frequent vomiting of a dark green, bilious matter; tongue very much coated with a thick yellow fur; constant thirst with a desire for cold drinks; pulse about 100, small and weak; skin warm and moist, and the countenance anxious and dejected. On examining the abdomen I found it a good deal distended and tympanitic, and slightly tender to the touch, and in the right groin a large hernial tumor. The history she gave of her case was, that twenty years previous, while in Scotland, she was assisting to take in some hay, and while in the act of raising a large forkful, she felt something give way in the right groin, and a few days after she discovered a small tumor there, which disappeared upon slight pressure, but had re-appeared after a long walk or a hard day's work ever since, but always disappeared on going to bed, relaxing the muscles and making gentle pressure upwards. About ten years since, while in Scotland, it became strangulated, but was relieved by purgatives and opiates. Latterly it had become more troublesome, coming down oftener, and being more difficult to return, as well as more painful when it was down. This morning, immediately after she arose, she was seized with the symptoms enumerated above, which have continued pretty much the same throughout the day, sometimes abating in severity a little and then returning more severely. Every attempt to swallow anything, even the blandest liquids, produces vomiting. The pain has been considerably abated by pretty large doses of the tinct. opii, which she has taken, and the application of warm fomentations to the tumor. I placed my patient in the proper position, and made use of the taxis for a short time, but very soon desisted in consequence of the pain it gave her. I directed her to have opium 1 grain, calomel gr. iij. every three hours, the warm fomentations to be continued to the tumor, and a turpentine liniment to the entire surface of the abdomen.

*Jan.* 10*th*, 10 A. M.—Patient passed a very good night, less vomiting and less pain. Gums touched by the calomel, pulse small and weak, skin bathed with warm perspiration, abdomen tympanitic but less painful to the touch, tongue less coated and countenance less dejected. I again tried the taxis, but was again obliged to desist in consequence of the pain it gave the patient. Directed a warm bath every 6 hours, the liniment and the fomentations to be continued in the intervals. Opium gr. 1 every four hours, without the calomel, and large injections of warm water every four hours, so as to distend the colon to its utmost extent.

*Jan.* 11*th*, 10 A. M.—Patient passed a very good night, passed some wind from the bowels but no feculent matter. All the symptoms are relieved, no pain, and scarcely any sickness of the stomach, mouth very sore, teeth all loose, tumor less tense and less painful to the touch, pulse fuller and stronger and the countenance somewhat cheerful. Continue treatment, with the addition of light nourishment. My patient continued to improve (excepting that she passed no feculent matter from the bowels) up to the morning of the 15th, when she was again seized with violent pain and vomiting, which soon became stercoracious. The pain commenced in the tumor, but soon extended over the entire abdomen, which soon became very tympanitic and very tender to the touch. In the evening I told her that nothing but an operation would relieve her, to which she said she would submit, if no better, by morning. I directed one grain of morphine every four hours, and left.

*Jan.* 16*th*, 10 A. M.—Countenance sunk and anxious, skin cold, pale, shrunken and covered with a greasy perspiration, pulse small and thready, patient unable to articulate so as to be heard, unless the ear is placed close to her mouth. Increased tympanitis, but less pain. She readily consented now to an operation, and Dr. D. J. McKibbin having been called in consultation, readily agreed with me as to the necessity of operating immediately as the only chance for the patient, but considered that almost desperate under the circumstances. We now gave her tinct. opii ʒij., and waited half an hour, and then placed her upon the table in proper position to operate. I commenced my incision immediately above the base of the tumor and carried it over it in the course of the femoral vein to a point about half an inch below it, and down to the superficial fascia of the thigh, which I pinched up and separated from the parts beneath by rubbing it between my thumb and finger, and made a slight opening into it and divided it upon the grooved director. I divided each successive layer in the same way till I came down to the sack, which was easily detected by the blue appearance and elastic feel of the distended intestine within it. I now passed my finger upon the outside of the sack and divided Gimbernat's ligament, when the stricture was immediately relieved, and the gas passed out of the strangulated intestine. I now attempted to return the sack and intestine entire, but found the sack adherent in every part so as to render that impossible. I then dissected the sack loose with my fingers and the handle of the scalpel, and attempted to return, but this I could not accomplish in consequence of another stricture at the neck of the sack. I now concluded to open the sack and divide the stricture at its neck, but this I found very difficult to accomplish in consequence of the intestine having contained nothing but gas, and this having escaped as soon as the stricture upon the outside of the sack was divided, and left it like a piece of wet bladder; but after a good deal of trouble I succeeded in separating the sack from the intestine, and puncturing it, then by seizing the whole and drawing it down, I was enabled to pass the director and divide the sack upon it. As soon as the sack was divided there was a discharge of about one and a half ounces of clear serum. The bowel appeared rather dark and congested, but otherwise healthy. I returned the bowel first and then the sack, and closed the wound by three stitches of the interrupted suture and straps of adhesive plaster. The patient was very much exhausted, but expressed herself entirely relieved. We now placed the patient in bed upon her back, with the right thigh flexed upon the pelvis in such a manner as to relax the abdominal muscles, and prevent any pressure upon the wound by the contents of the abdomen, and applied the simple warm water dressing. To have opium one gr. every four hours, with light broth and wine whey for nourishment. To be kept perfectly quiet and from changing her position.

*Jan. 17th*, 10 A. M.—Patient passed a very good night, no pain, very little thirst, gums less sore, tongue cleaning off, skin warm and moist, pulse one hundred, full and soft, countenance natural, less tympanites, and less soreness upon pressure. Passes wind freely from the bowels, but no feculent matter. Continue treatment as before.

*Jan. 18th*, 10 A. M.—Patient continues to improve; relishes nourishment, but still has passed no feculent matter. Continue treatment, with the addition of an occasional injection of warm water.

*Jan. 19th*, 10 A. M.—Patient continues to improve, wound nearly united by the first intention. Bowels have been freely moved by the injection; stool natural and consistent. From this time forward my patient continued to improve rapidly, and in about one month after she was operated upon she had entirely recovered. I saw her three days since, and she is in robust health, and the hernia seems to be radically cured, the cicatrix being firmly united to the pubic bone. She experiences no inconvenience from it whatever. The prominent points in this case are: 1st. The complete relief of all the symptoms of strangulation produced by calomel and opium and the soothing treatment, although the stricture was not removed. 2d. The hopelessness of any attempt at reduction, by the taxis, of an old hernia, although the patient may tell you, as mine did, that the tumor only came down that morning, for I am satisfied, from the amount of adhesions, that the hernia had been irreducible for the last ten years. 3d. The injury that would result from persisting in the taxis in such a case, the use of tart. ant., the tobacco injection, and the many other remedies that are recommended in the books, in strangulated hernia. And lastly, the complete success of an operation under circumstances apparently so hopeless.

CASE II.—DISLOCATION OF THE FEMUR.

I was called to see Samuel B., November 22d, 1850, and on enquiry I found that some two hours previous he had been mining, upon his knees, when a fall of coal came down and struck him upon the back part of the pelvis, in such a manner as to force the head of the femur out of its socket and place it upon the dorsum of the ilium, presenting outwards and backwards, with the great trochanter resting against the edge of the acetabulum behind, the limb being shortened near three inches, the foot inverted and resting upon the other above the instep. I had purchased Dr. Jarvis's instrument a few days previous, and I thought this a very good opportunity for testing its good qualities; so I placed my patient upon a common deal table, and applied the instrument according to the Doctor's directions; and now I supposed that all I had to do was to turn the ratchet and the bone would fly to its place; but after torturing my patient for some two hours, trying my utmost to make the extension in the line of displacement, I gave it up in despair, and adjourned the case till nine o'clock the next day, very much cooled in regard to the infallibility of the instrument I had purchased, at least in my hands. But still supposing that my failure was more owing to my inability to apply it than to any fault in the instrument, I sent a note to Dr. Carpenter, (upon whose recommendation I had been induced to purchase the instrument,) a gentleman of acknowledged abilities in all cases of the kind, asking him to meet me in consultation in the case. We met punctually to the time, and I again applied the instrument, and asked him to take charge of the limb and reduce it. He took charge of the limb, and, laboring for near three hours, he gave it up in despair, also perfectly satisfied that he could not make the extension in the line of displacement, (the very thing for which the inventor recommends it so highly,) and that the lever placed upon the instrument would make a very nice plaything for a child, but would certainly never subserve the purpose for which it was intended, viz., forcing the head of the bone out of its malposition. We now put the patient under the influence of chloroform, and applied the sheets in the old way recommended in the books, but soon found that we were unable to make extension sufficiently long to overcome the resistance of the muscles; consequently we adjourned the case until 3 o'clock P. M., when we again met and applied the compound pulley, and put the patient under the influence of chloroform, when it was reduced in twenty minutes. The patient was placed comfortably in bed, and at the end of a week had so far recovered as to walk about with ease. I would say in this connection to all medical students, beware of all patented instruments for setting fractures and reducing luxations, and in their place get a few good splints and a set of compound pulleys, as they alone can be relied on in practice, notwithstanding the many certificates they possess from many of our professors who stand deservedly high in the profession.

CASE III.—DISLOCATION OF THE FEMUR.

I was called in consultation with a neighboring physician to Dennis D., a young man about twenty-five years of age, a miner by occupation. Some four hours previous, while engaged in mining, upon his knees, a fall of coal came down, and a large piece with a sharp cutting edge struck him immediately over the os coccyx, cutting the soft parts down to the bone, passing over the sphincter ani muscle, which receded before the force of the blow and was uninjured; it passed through the entire length of the perineum and through the scrotum, separating the testicles, leaving one upon either side of the incision. The depth of the incision was about one inch. I cleaned out the wound by removing the coal dirt and other foreign substances, and closed it with the interrupted suture and adhesive straps, and ordered the simple warm water dressing. Not supposing he had received any other injury, as he made no other complaint, I made no further examination, but left him in the hands of his attending physician. On the 8th of January, 1852, just seven weeks after he had been injured, I was again called to see him, and was informed that he had not yet been able to leave his bed, and that he had dismissed his physician because he was dissatisfied with his attendance. On examination, a dislocation of the femur upwards and backwards into the sciatic notch was discovered, which had occurred at the time of his injury. The wound of the perineum was completely cicatrized, and I must confess that I felt mortified that I had overlooked the dislocation at my first visit, as I knew the attending physician, who was quite a young man, depended upon me for a proper diagnosis in the case, and as I had failed to detect it he had never suspected such a thing. Seeing that the man must remain a cripple for life if the dislocation remained unreduced, and that my reputation must suffer as a necessary consequence, I determined, notwithstanding the length of time it had remained in its malposition, to attempt its reduction at all hazards.

On the 9th of January, in consultation with Dr. McKibbin, we placed the patient on a bed upon the floor, and applied the compound pulleys with the counter extending band upon the injured side, and kept up extension for about 20 or 25 minutes at a time, with intervals of rest for about two hours, without being able to dislodge the head of the bone from the position it had taken. We then shifted the counter extending band to the sound side, and after keeping up extension constantly for about half an hour, the head of the bone gradually slipped into its place without the slightest noise. So gradual had been its reduction that we were not satisfied that it was reduced until, in order to satisfy ourselves, we commenced flexing and extending the limb when it slipped out again. We again applied the pulleys and reduced it with a very slight effort; and again, in trying to satisfy ourselves of its proper reduction, it slipped out, but was again reduced with a very slight effort; when we placed the patient upon his back in bed and bound his knees together with a strong roller, and kept him in that position for four weeks, when he was permitted to get up and go about on crutches. He has at this date entirely recovered the use of his limb. The acetabulum appeared to be almost entirely filled, hence the ease with which it slipped out of its place after reduction. That the head of the bone occupied the sciatic notch was clearly demonstrated by an examination through the rectum, and by the canal left in the fibres of the gluteic muscles after its reduction from that point to the acetabulum, caused by its having traversed from one point to the other.

The prominent points in this case are: 1st. The necessity of a thorough examination of every case to which we are called, and the great injury that may result, not only to the patient, but to our own reputation and to the profession at large, from its neglect. 2d. That a dislocated femur may be reduced, nearly two months after the accident, without injury to the patient; and 3d. The necessity of keeping up extension and counter-extension continuously till the resistance of the muscles are overcome, or while the strength of the patient will permit, before giving up our effort.

